# Supplementary material for: Morphological and Physiological Mechanisms of Melatonin on Delaying Drought-Induced Leaf Senescence in Cotton
Source: Int J Mol Sci. 2023 Apr 14;24(8):7269. doi: 10.3390/ijms24087269 (PMC10138977; doi:10.3390/ijms24087269)
Supplement: Supplementary file 1 [file ijms-24-07269-s001.zip › ijms-2305429-Supplementary.pdf]

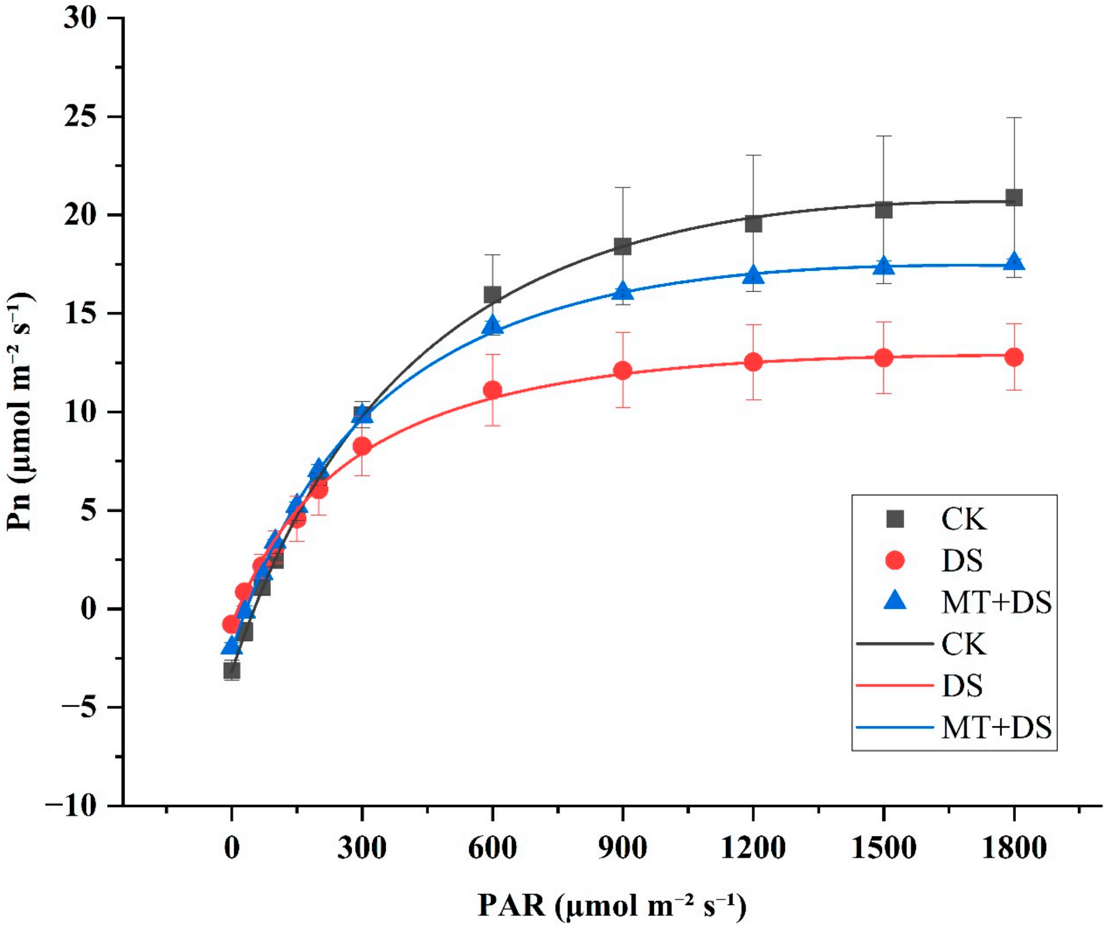

Figure S1. Effects of melatonin on the light response curve of cotton seedlings during drought-induced senescence. CK denotes well-watered + 0 μM MT treatment; DS denotes drought stress + 0 μM MT treatment; MT+DS denotes drought stress + 100 μM MT treatment.

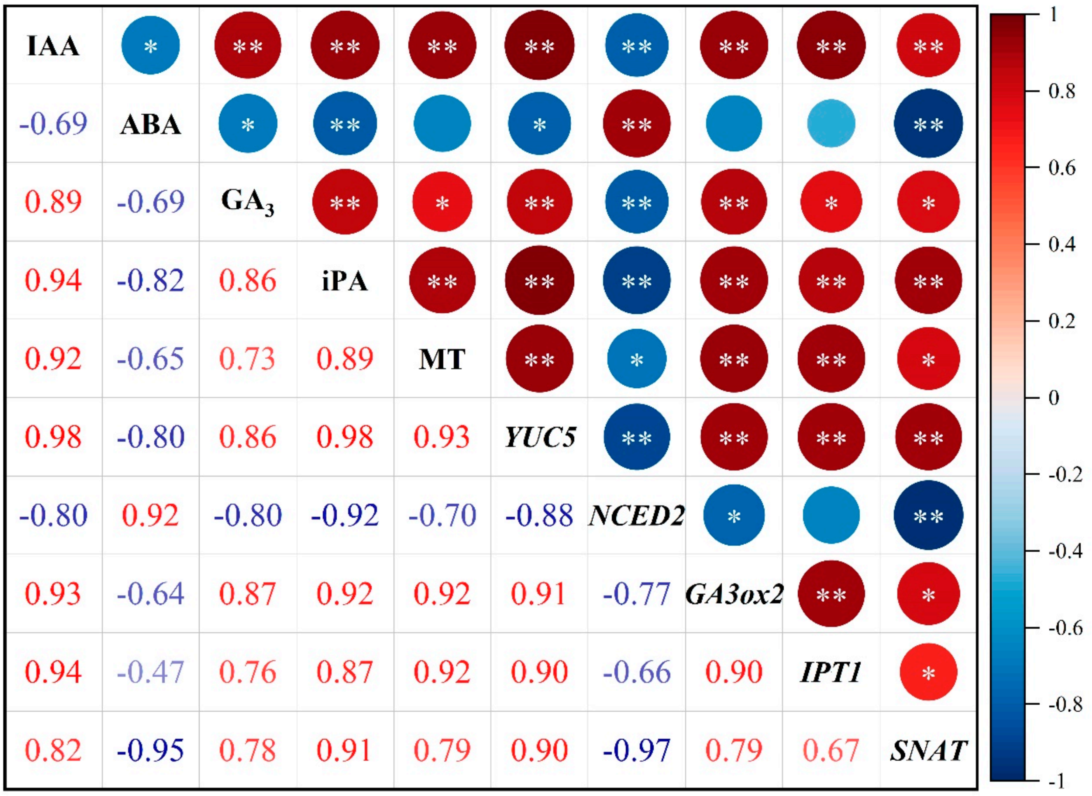

Figure S2. Pearson's correlation coefficient showing the relationship between endogenous hormone content and key genes of hormone synthesis. The significance level of correlations is indicated as * denoting $p < 0.05$ or ** denoting $p < 0.01$.

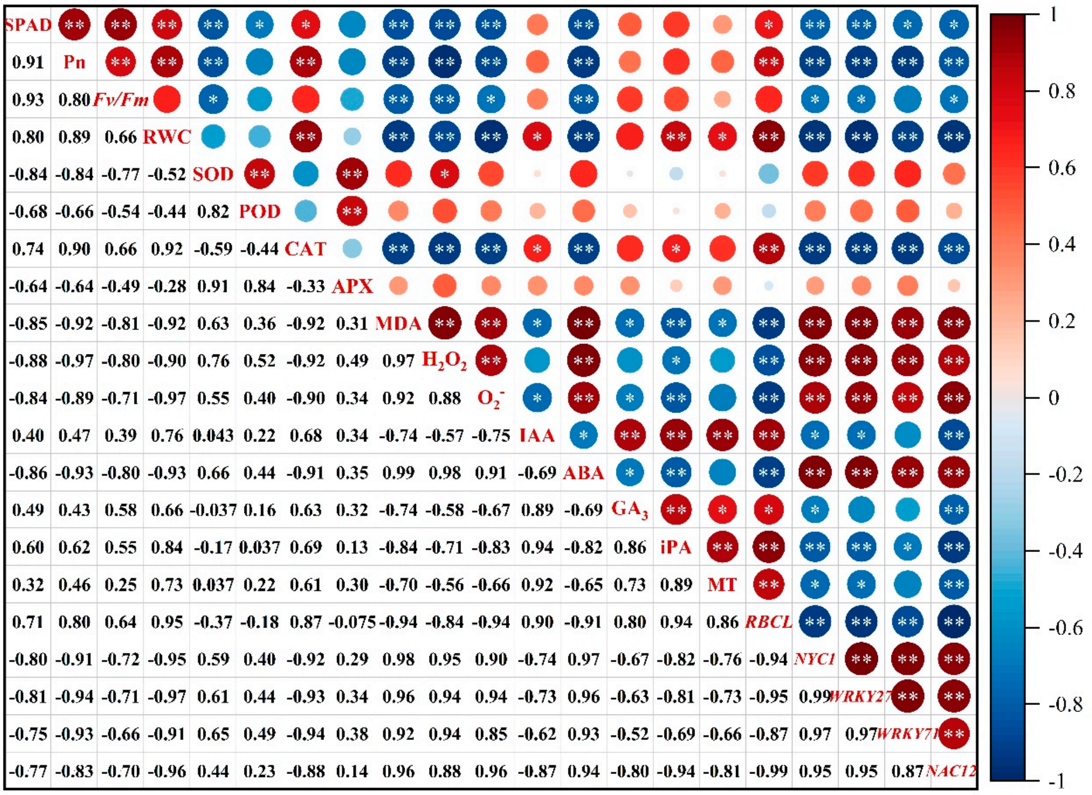

Figure S3. The Pearson correlation coefficients matrix and 95% confidential circle among the physiological indicators and senescence-related gene expression in cotton seedlings. The significance level of correlations is indicated as * denoting $p < 0.05$ or ** denoting $p < 0.01$. SPAD: spad value; Pn: net photosynthetic rate; Fv/Fm: maximum photochemical efficiency; RWC: relative water content; SOD: superoxide dismutase activity; POD: peroxidase activity; CAT: catalase activity; APX: ascorbate peroxidase activity; MDA: malonaldehyde contents; H_2O_2: H_2O_2 contents; O_2^-: O_2^- contents; IAA: auxin contents; ABA: abscisic acid contents; GA_3: gibberellin acid contents; iPA: isopentenyladenine contents; MT: melatonin contents; *RBCL*: transcript levels of *GhRBCL*; *NYC1*: transcript levels of *GhNYC1*; *WRKY27*: transcript levels of *GhWRKY27*; *WRKY71*: transcript levels of *GhWRKY71*; *NAC12*: transcript levels of *GhNAC12*.

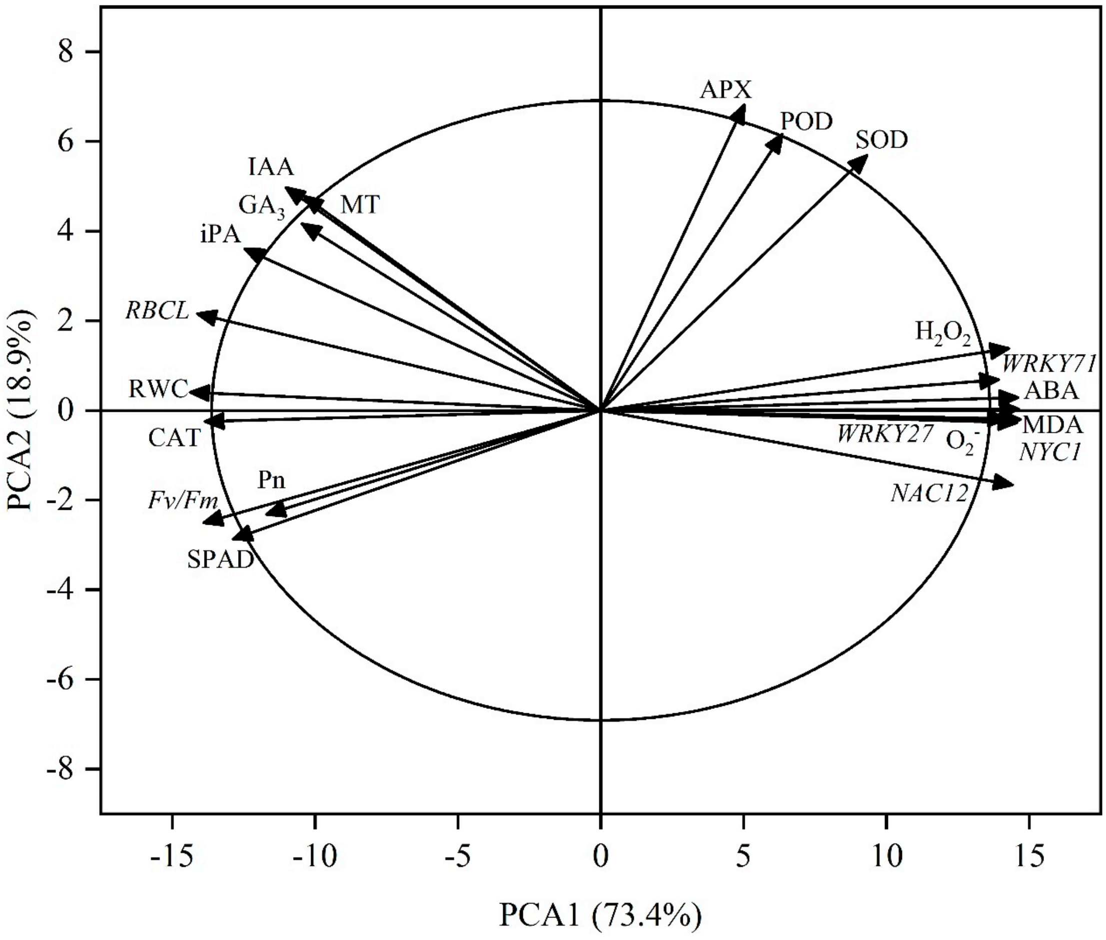

Figure S4. Principal component analysis (PCA) of the 21 representative leaf senescence traits (abbreviations are as expounded in Fig. S3).
